# Supplementary material for: Ethics and legal requirements for data linkage in 14 European countries for children with congenital anomalies
Source: BMJ Open. 2023 Jul 27;13(7):e071687. doi: 10.1136/bmjopen-2023-071687 (PMC10387628; doi:10.1136/bmjopen-2023-071687)
Supplement: Author's manuscript [file bmjopen-2023-071687.draft_revisions.pdf]

BMJ Open is committed to open peer review. As part of this commitment we make the peer review history of every article we publish publicly available.

When an article is published we post the peer reviewers' comments and the authors' responses online. We also post the versions of the paper that were used during peer review. These are the versions that the peer review comments apply to.

The versions of the paper that follow are the versions that were submitted during the peer review process. They are not the versions of record or the final published versions. They should not be cited or distributed as the published version of this manuscript.

BMJ Open is an open access journal and the full, final, typeset and author-corrected version of record of the manuscript is available on our site with no access controls, subscription charges or pay-per-view fees (http://bmjopen.bmj.com).

If you have any questions on BMJ Open's open peer review process please email info.bmjopen@bmj.com

BMJ Open

Ethics and legal requirements for data linkage in 14 European countries

Journal:	*BMJ Open*
Manuscript ID	bmjopen-2023-071687.R1
Article Type:	Communication
Date Submitted by the Author:	22-Jan-2023
Complete List of Authors:	Claridge, Hugh; University of Surrey, School of Health Sciences<br>Tan, Joachim; St George's University of London, Population Health Research Institute<br>Loane , Maria ; Ulster University Faculty of Life and Health Sciences<br>Garne, Ester; Lillebaelt Hospital - University Hospital of Southern Denmark, Department of Paediatrics and Adolescent Medicine<br>Barisic, Ingeborg; Children's Hospital Zagreb, Centre of Excellence for Reproductive and Regenerative Medicine, Medical School University of Zagreb<br>Cavero- Carbonell, Clara; Foundation for the Promotion of Health and Biomedical Research in the Valencian Region, Rare Diseases Research Unit<br>Dias, Carlos; National Institute of Health Doctor Ricardo Jorge, Epidemiology Department, National Registry of Congenital Anomalies<br>Gatt, Miriam; Directorate for Health Information and Research, Malta Congenital Anomalies Registry<br>Jordan, Susan; Swansea University, Faculty of Medicine, Health & Life Sciences<br>Khoshnood, Babak; University of Paris, Epidemiology and Statistics Research Center, Obstetrical, Perinatal and Pediatric Epidemiology Research Team, Institut National de la Santé et de la Recherche Médicale<br>Kiuru-Kuhlefelt, Sonja; Finnish Institute for Health and Welfare, Knowledge Brokers<br>Klungsoyr, Kari; University of Bergen, Department of Global Public Health and Primary Care; Norwegian Institute of Public Health, Divison of Mental and Physical Health<br>Mokoroa Carollo, Olatz; Biodonostia Health Research Institute, Public Health Division of Gipuzkoa<br>Nelen, Vera; Provincial Institute for Hygiene<br>Neville, Amanda; University of Ferrara, IMER Registry<br>Pierini, Anna; Institute of Clinical Physiology National Research Council<br>Randrianaivo, Hanitra; Centre Hospitalier Universitaire de la Reunion, Register of Congenital Malformations<br>Rissmann, Anke; Otto von Guericke University Medical Faculty, Malformation Monitoring Centre Saxony-Anhalt<br>Tucker, David; Public Health Wales NHS Trust<br>de Walle, Hermien; University of Groningen, University Medical Center Groningen, Department of Genetics<br>Wertelecki, Wladimir; OMNI-Net for Children International Charitable Fund<br>Morris, Joan; St George's University of London, Population Health

	Research Institute
**Primary Subject Heading**:	Ethics
Secondary Subject Heading:	Epidemiology, Public health
Keywords:	ETHICS (see Medical Ethics), EPIDEMIOLOGY, PUBLIC HEALTH, REGISTRIES

I, the Submitting Author has the right to grant and does grant on behalf of all authors of the Work (as defined in the below author licence), an exclusive licence and/or a non-exclusive licence for contributions from authors who are: i) UK Crown employees; ii) where BMJ has agreed a CC-BY licence shall apply, and/or iii) in accordance with the terms applicable for US Federal Government officers or employees acting as part of their official duties; on a worldwide, perpetual, irrevocable, royalty-free basis to BMJ Publishing Group Ltd ("BMJ") its licensees and where the relevant Journal is co-owned by BMJ to the co-owners of the Journal, to publish the Work in BMJ Open and any other BMJ products and to exploit all rights, as set out in our licence.

The Submitting Author accepts and understands that any supply made under these terms is made by BMJ to the Submitting Author unless you are acting as an employee on behalf of your employer or a postgraduate student of an affiliated institution which is paying any applicable article publishing charge ("APC") for Open Access articles. Where the Submitting Author wishes to make the Work available on an Open Access basis (and intends to pay the relevant APC), the terms of reuse of such Open Access shall be governed by a Creative Commons licence – details of these licences and which Creative Commons licence will apply to this Work are set out in our licence referred to above.

Other than as permitted in any relevant BMJ Author's Self Archiving Policies, I confirm this Work has not been accepted for publication elsewhere, is not being considered for publication elsewhere and does not duplicate material already published. I confirm all authors consent to publication of this Work and authorise the granting of this licence.

For peer review only

TITLE: Ethics and legal requirements for data linkage in 14 European countries

AUTHORS

Hugh Claridge, School of Health Sciences, University of Surrey, UK

Joachim Tan, Population Health Research Institute, St George's, University of London, UK

Maria Loane, Faculty of Life and Health Sciences, Ulster University, Belfast, UK

Ester Garne, Department of Paediatrics and Adolescent Medicine, Lillebaelt Hospital, University Hospital of Southern Denmark, Kolding, Denmark

Ingeborg Barisic, Children's Hospital Zagreb, Centre of Excellence for Reproductive and Regenerative Medicine, Medical School University of Zagreb, Croatia

Clara Cavero-Carbonell, Rare Diseases Research Unit, Foundation for the Promotion of Health and Biomedical Research in the Valencian Region (FISABIO), Valencia, Spain

Carlos Dias, Epidemiology Department, National Registry of Congenital Anomalies, National Institute of Health Doutor Ricardo Jorge (Instituto Nacional de Saúde Doutor Ricardo Jorge), Lisbon, Portugal

Miriam Gatt, Malta Congenital Anomalies Registry, Directorate for Health Information and Research, Pieta, Malta

Susan Jordan, Faculty of Medicine, Health & Life Sciences, Swansea University, Swansea, Wales

Babak Khoshnood, Université de Paris, CRESS-Epopé, INSERM, INRA, Paris, France

Sonja Kiuru-Kuhlefelt, Knowledge Brokers, Finnish Institute for Health and Welfare, Helsinki, Finland

Kari Klungsoyr, Department of Global Public Health and Primary Care, University of Bergen and Division of Mental and Physical Health, Norwegian Institute of Public Health, Bergen, Norway

Olatz Mokoroa Carollo, Public Health Division of Gipuzkoa, BioDonostia Research Institute, San Sebastian, Spain

Vera Nelen, Provincial Institute for Hygiene, Antwerp, Belgium

Amanda J Neville, Registro IMER, University of Ferrara, Ferrara, Emilia-Romagna, Italy

Anna Pierini, Institute of Clinical Physiology National Research Council, Pisa, Italy

Hanitra Randrianaivo, Register of Congenital Malformations, Centre Hospitalier Universitaire de La Réunion, Île de la Réunion, France

Anke Rissmann, Malformation Monitoring Centre Saxony-Anhalt, Medical Faculty Otto-von-Guericke University, Magdeburg, Germany

David Tucker, Public Health Wales National Health Service Trust, Wales, United Kingdom

Hermien de Walle, University of Groningen, University Medical Center Groningen, Department of Genetics, Groningen, the Netherlands

For peer review only - http://bmjopen.bmj.com/site/about/guidelines.xhtml

Wladimir Wertelecki, OMNI-Net for Children International Charitable Fund, Rivne, Ukraine

Corresponding author: Joan K. Morris, Population Health Research Institute, St George's, University of London, London, UK, SW17 0RE jmorris@sgul.ac.uk

WORD COUNT

1788 words

KEYWORDS

Ethics; Information Storage and Retrieval; Methods; Congenital Abnormalities; Rare Diseases; Europe

ABSTRACT

Introduction

Linking health care datasets can create valuable resources for research, particularly when investigating rare exposures or outcomes. However, across Europe the permissions processes required to access data can be complex. This paper documents the processes required by the EUROlinkCAT study investigators to research the health and survival of children with congenital anomalies in Europe.

Methods

Eighteen congenital anomaly registries in 14 countries provided information on all the permissions required to perform surveillance of congenital anomalies and to link their data on live births with available vital statistics and health care databases for research. Small number restrictions imposed by data providers were also documented.

Results

The permissions requirements varied substantially, with certain registries able to conduct congenital anomaly surveillance as part of national or regional health care provision, whilst others were required to obtain ethics approvals or informed consent. Data linkage and analysis for research purposes added additional layers of complexity for registries, with some required to obtain several permissions, including ethics approvals to link the data. Restrictions relating to small numbers often resulted in a registry's data on specific congenital anomalies being unusable.

Conclusion

The permissions required to obtain and link data on children with congenital anomalies varied greatly across Europe. The variation and complexity present a significant obstacle to the use of such data, especially in large data linkage projects. Furthermore, small number restrictions severely limited the research that could be performed for children with specific rare congenital anomalies.

For peer review only - http://bmjopen.bmj.com/site/about/guidelines.xhtml

INTRODUCTION

The EUROlinkCAT study aimed to support 22 congenital anomaly registries in 14 countries to link their data on live births with congenital anomalies to other regional or national electronic health care databases to investigate the survival and morbidity of these children up to ten years of age. This article briefly summarises the different legal, ethical, governance and other permissions that the registries had to obtain to perform this work.

METHODS

Each congenital anomaly registry was required to submit to the Coordinating Beneficiary of EUROlinkCAT (St George's, University of London), the permissions for the registry to fulfil its surveillance remit and to send data to EUROCAT (the European network of population-based congenital anomaly registries). An umbrella protocol was then supplied to each registry providing sufficient information for them to apply to the relevant authorities to enable them to link their data on liveborn children with congenital anomalies to national and regional electronic databases containing information on the survival and morbidity of these children. The registries were responsible for obtaining the necessary permissions, and once obtained these were submitted to the EUROlinkCAT Coordinator. This article summarises all the permissions obtained.

The English data on congenital anomalies included in the EUROlinkCAT project were provided by five regional congenital anomaly registries. However, in April 2015, the regional registries in England were all combined into the National Congenital Anomaly and Rare Disease Registration Service (NCARDRS). As the regional registries no longer exist, the permissions that would be required to obtain data from NCARDRS are reported here instead. This is why information is available for 18 registries in total, rather than the original 22.

Information from 18 registries in 14 countries are presented, relating to the timeframe of the EUROlinkCAT project from 2017-2022 – it is therefore possible that some permission requirements have since changed.

The answers from the registries were categorised in Table 1 under the following headings:

1. Permissions required for registry to perform surveillance of congenital anomalies
    - 1.1. Permission granted as part of regional/national health care provision
        - 1.1.1. Yes: National or regional legislation exists that enables the registry to collect data for surveillance purposes
        - 1.1.2. No: National or regional legislation does not exist to enable the registry to collect data for surveillance purposes
    - 1.2. Permissions from hosting institution
        - 1.2.1. Yes: The registry needs to obtain permission to collect data for surveillance
        - 1.2.2. No: No additional permission is needed as national/regional legislation is sufficient

For peer review only - http://bmjopen.bmj.com/site/about/guidelines.xhtml

1.3. Ethics permission

1.3.1. Yes: The registry needs to obtain ethics permission to collect data for surveillance

1.3.2. No: The registry does not need to obtain ethics permission to collect data for surveillance

1.4. Informed consent needed

1.4.1. Yes: The registry needs to obtain informed consent from the parents to register the congenital anomaly

1.4.2. No: The registry does not need to obtain informed consent from the parents to register the congenital anomaly. However, the parents can choose to have their details removed by contacting the relevant organisation.

2. Permissions required for registry to link data and analyse for research purposes

2.1. Ethics

2.1.1. Yes: Ethics permission is needed for research projects to link and analyse the linked data

2.1.2. No: Ethics permission is not needed for research projects using the linked data

2.2. Other permissions needed

2.2.1. Yes: Other permissions, not including ethics, such as approval by the data provider regarding the safety of the data storage, permissions to share the data with other named parties or permissions to release the analytic results for publication are required

2.2.2. No: No additional permissions are needed for research using the linked data

2.3. Small number restrictions

2.3.1. Yes-Pub: Small number restrictions apply to any publication of the data, but pre-publication results provided to named researchers may include small numbers

2.3.2. Yes-Int: Small number restrictions apply to all use of data including pre-publication results

2.3.3. No: No small number restrictions need to be applied.

For peer review only

For peer review only - http://bmjopen.bmj.com/site/about/guidelines.xhtml

RESULTS

Table 1: The permissions required for congenital anomaly registries to perform surveillance and research.

Full name of CA Registry	Geographical Region	Permissions required for registry to perform surveillance of congenital anomalies				Permissions required for registry to link data and analyse for research purposes			Notes
		Permission granted as part of regional/national health care provision	Permission from hosting institution	Ethics permission	Informed consent	Ethics permission	Other Permissions	Small Number Restric-tions	
**Antwerp Congenital Anomaly Registry**	Belgium: Antwerp	Yes	No	No	No	Yes	Yes	Yes-Int	Considered linkage to mortality data only
**Zagreb Congenital Anomaly Registry**	Croatia: Zagreb	No	Yes	No	No	Yes	No	No	
**Odense**	Denmark: Funen	Yes	Yes	No	No	No	Yes	Yes-Pub	
**Finnish Register of Congenital Malformations**	Finland: all	Yes	No	No	No	Yes	No	No	

For peer review only - http://bmjopen.bmj.com/site/about/guidelines.xhtml

Full name of CA Registry	Geographical Region	Permissions required for registry to perform surveillance of congenital anomalies				Permissions required for registry to link data and analyse for research purposes			Notes
		Permission granted as part of regional/national health care provision	Permission from hosting institution	Ethics permission	Informed consent	Ethics permission	Other Permissions	Small Number Restric-tions	
**Registre des malformations congénitales de l'île de la Réunion**	France: Île de la Réunion	Yes	No	Yes	No	Yes	Yes	No	
**Registre des malformations congénitales de Paris**	France: Paris	Yes	No	Yes	No	Yes	Yes	No	Considered linkage to mortality data only
**Saxony-Anhalt Congenital Anomaly Registry**	Germany: Saxony-Anhalt	No	No	Yes	Yes	Yes	No	No	Manual linkage only to mortality data due to Bundesstatistikgesetz $21 (Prohibition of re-identification)

For peer review only - http://bmjopen.bmj.com/site/about/guidelines.xhtml

Full name of CA Registry	Geographical Region	Permissions required for registry to perform surveillance of congenital anomalies				Permissions required for registry to link data and analyse for research purposes			Notes
		Permission granted as part of regional/national health care provision	Permission from hosting institution	Ethics permission	Informed consent	Ethics permission	Other Permissions	Small Number Restric-tions	
**IMER Registry**	Italy: Emilia Romagna	Yes	No	No	No	No	Yes	No	
**Tuscany Congenital Anomaly Registry**	Italy: Tuscany	Yes	No	No	No	No	Yes	No	
**Malta Congenital Anomaly Registry**	Malta: all	Yes	No	No	No	Yes	No	No	Considered linkage to mortality data only
**EUROCAT Registry - Northern Netherlands**	Netherlands: Northern	Yes	No	No	Yes	Yes	Yes	Yes-Int	Other permissions: parents are given the option to opt out of EUROlinkCAT

For peer review only

Full name of CA Registry	Geographical Region	Permissions required for registry to perform surveillance of congenital anomalies				Permissions required for registry to link data and analyse for research purposes			Notes
		Permission granted as part of regional/national health care provision	Permission from hosting institution	Ethics permission	Informed consent	Ethics permission	Other Permissions	Small Number Restric-tions	
**Medical Birth Registry of Norway**	Norway: All	Yes	No	No	No	Yes	Yes	No	Considered linkage to mortality data only
**RENAC: Registo Nacional de Anomalias Congénitas**	Portugal: South	Yes	Yes	No	No	Yes	Yes	No	
**Basque Congenital Anomaly Registry**	Spain: Basque Country	Yes	No	No	No	Yes	No	No	
**Valencian Region Congenital Anomalies Registry**	Spain: Valencian Region	Yes	No	No	No	Yes	No	No	

For peer review only - http://bmjopen.bmj.com/site/about/guidelines.xhtml

Full name of CA Registry	Geographical Region	Permissions required for registry to perform surveillance of congenital anomalies				Permissions required for registry to link data and analyse for research purposes			Notes
		Permission granted as part of regional/national health care provision	Permission from hosting institution	Ethics permission	Informed consent	Ethics permission	Other Permissions	Small Number Restric-tions	
**National Congenital Anomaly and Rare Disease Registration Service (NCARDRS)**	UK: England	Yes	No	No	No	Yes	Yes	Yes-Pub	NCARDRS became part of NHS Digital in October 2021 and access conditions are currently under review
**Congenital Anomaly Register and Information Service (CARIS) linked with SAIL databank**	UK: Wales	Yes	No	Yes	No	No	Yes	Yes-Pub	
**OMNI-Net Ukraine Birth Defects Program**	Ukraine: West	Yes	No	No	No	No	No	No	**Considered linkage to**

For peer review only - http://bmjopen.bmj.com/site/about/guidelines.xhtml

Full name of CA Registry	Geographical Region	Permissions required for registry to perform surveillance of congenital anomalies				Permissions required for registry to link data and analyse for research purposes			Notes
		Permission granted as part of regional/national health care provision	Permission from hosting institution	Ethics permission	Informed consent	Ethics permission	Other Permissions	Small Number Restric-tions	
									**mortality data only**

Yes-Pub: Small number restrictions apply to any publication of the data, but pre-publication results to named researchers may include small numbers
Yes-Int: Small number restrictions apply to all use of data including pre-publication results.

peer review only

For peer review only - http://bmjopen.bmj.com/site/about/guidelines.xhtml

Permissions required for surveillance of congenital anomalies

The majority (16/18) of registries have permission to perform surveillance of congenital anomalies as part of their national or regional health care provision. Of these 16, three are required to obtain ethics approval to perform this surveillance and one is required to obtain informed consent from the parents/guardians of the children. Of the two registries that did not have permission under their national or regional health care provision, one needed permission to collect the data from their host institution and one needed both ethics permission and informed consent.

Permissions required for data linkage and analysis for research purposes

Of the six registries who only linked their data to mortality data, five required specific ethics permission to do so, and three of these also required other additional permissions. Of the 12 registries who linked their data to electronic health care databases, eight required specific ethics permission to do so and four of these required other additional permissions. The four registries who did not require specific ethics permission to link their data to electronic health care databases did however require other additional permissions, such as demonstrating to the data providers that the data would be stored safely, that the project was of sufficient scientific merit, and other assurances arising from the European Union's General Data Protection Regulation (GDPR) 2016/679 requiring that the data be used in a transparent manner.

Once the data had been linked to other databases, the authorities providing these data often imposed strict restrictions on the publication of results when small numbers of children were involved. This was to ensure that children with rare congenital anomalies and their families could not be identified. Some authorities did allow aggregate results from small numbers of individuals to be distributed amongst named researchers with the requirement that these results could only be published when combined with data from other registries. Permitting the internal distribution amongst specified researchers was extremely helpful as it enabled meta-analyses over several European countries to be performed for groups of rare congenital anomalies and combined results to be published. Authorities suppressing the provision of small numbers of cases for analysis (e.g. through rounding counts to the nearest 5) often resulted in a registry's data being excluded from the meta-analyses due to the rounding having a significant effect on the overall estimates.

The length of time required to obtain the necessary permissions varied considerably by registry, with several registries taking over two years to obtain the permissions and thus over two years to obtain the final linked data. In contrast, some institutions granted approvals within three months.

DISCUSSION

To the authors' knowledge, this is the first example in the literature of a multi-centre, multi-national health data linkage project documenting the broad range of permissions required across Europe to access and link data on children with congenital anomalies. The increased awareness that this article provides of the high variability in data providers' requirements and the timescales that the

For peer review only - http://bmjopen.bmj.com/site/about/guidelines.xhtml

permissions-related processes operate within, will be of great assistance to funding bodies and both current and future studies in this and related fields. For multi-centre studies, the widely variable waiting times make it very challenging to determine a common timeframe for the overall study to work to. Indeed, the challenges faced by EUROlinkCAT researchers are not unique, with researchers across the world facing similar issues (UK:[1]; Australia:[2]; USA:[3]). Advanced knowledge of the challenges means staff time and thus personnel budgets can be more accurately costed over a project's timeframe; awareness of the possibility of permissions inter-dependencies can permit improved planning and more efficient task prioritisation; data access expiration dates can be more appropriately chosen to limit the need for amendments and re-applications; and staff can be better prepared for the sometimes demoralising wait for permissions to be granted.

The suppression of small numbers, such as preventing the release of numbers smaller than 10 even for internal analysis purposes, has a seriously detrimental impact on rare disease research. To illustrate this problem, consider Ebstein's Anomaly, which is a severe rare congenital heart defect with a mortality of around 50% in the first month of life,[4] and continued high mortality in the first five years of life.[5] The prevalence of Ebstein's Anomaly is about 4 per 100,000 births.[6] EUROlinkCAT registries have survival information on about 400 live births with Ebstein's Anomaly. If the numbers of children dying during the first month of life, the numbers dying after the first month of life and before their first birthday, and the numbers surviving past the age of one are required, all 22 EUROlinkCAT registries would be likely to have at least one out of the three cells below 10 and therefore none of the registries would be able to provide any aggregated data. Hence, no information about mortality during the first year of life could be obtained, despite a sample size of 400 children from a study period of 20 years and a population of 10 million births.

The rationale for small number restrictions is to prevent individuals or their families being identified. A distinction needs to be made between data released for publication and data released to named researchers for analysis. The implementation of GDPR across Europe in 2018 has ensured that all European countries have structures in place to ensure the safe use of an individual's data. These regulations should be applied in such a way as to allow all European countries to provide aggregate data to be used for pan-European analyses across countries to trusted, named researchers, even if certain categories of such aggregate data comprise fewer than 10 cases.

CONCLUSIONS

There is a lack of consistency across Europe in the permissions required to access and link data on children with congenital anomalies. The complexity and time taken to obtain such permissions present a significant obstacle to the use of such data. In addition, the imposition of small number restrictions has a detrimental effect on the analyses that can be performed for children with rare anomalies.

For peer review only - http://bmjopen.bmj.com/site/about/guidelines.xhtml

AUTHORS' CONTRIBUTIONS

JKM is the principal author and the project's scientific coordinator. HC is the senior project manager. JT is the senior statistician. ML is the data coordinator. EG is the clinical coordinator and EUROCAT registry leader. VN, IB, SK-K, HR-R, BK, AR, AJN, AP, MG, HEKdW, KK, CMD, OMC, CC-C, DFT, SJ and WW are EUROCAT registry leaders. All authors contributed to, read and approved the final manuscript.

FUNDING STATEMENT

This project has received funding from the European Union's Horizon 2020 Research and Innovation programme under grant agreement No 733001 for 5 years: 1 January 2017 to 31 May 2022. The views presented here are those of the authors only, and the European Commission is not responsible for any use that may be made of the information presented here.

COMPETING INTERESTS STATEMENT

The authors have no competing interests to declare.

For peer review only

REFERENCES

1 Taylor JA, Crowe S, Pujol FE, *et al.* The road to hell is paved with good intentions: the experience of applying for national data for linkage and suggestions for improvement. *BMJ Open* 2021;**11**:e047575. doi:10.1136/bmjopen-2020-047575

2 Palamuthusingam D, Johnson DW, Hawley C, *et al.* Health data linkage research in Australia remains challenging. *Internal Medicine Journal* 2019;**49**:539–44. doi:10.1111/imj.14244

3 Bradley CJ, Penberthy L, Devers KJ, *et al.* Health Services Research and Data Linkages: Issues, Methods, and Directions for the Future. *Health Services Research* 2010;**45**:1468–88. doi:10.1111/j.1475-6773.2010.01142.x

4 McElhinney DB, Salvin JW, Colan SD, *et al.* Improving Outcomes in Fetuses and Neonates With Congenital Displacement (Ebstein's Malformation) or Dysplasia of the Tricuspid Valve. *The American Journal of Cardiology* 2005;**96**:582–6. doi:10.1016/j.amjcard.2005.04.024

5 Holst KA, Dearani JA, Said SM, *et al.* Surgical Management and Outcomes of Ebstein Anomaly in Neonates and Infants: A Society of Thoracic Surgeons Congenital Heart Surgery Database Analysis. *The Annals of Thoracic Surgery* 2018;**106**:785–91. doi:10.1016/j.athoracsur.2018.04.049

6 Boyle B, Garne E, Loane M, *et al.* The changing epidemiology of Ebstein's anomaly and its relationship with maternal mental health conditions: a European registry-based study. *Cardiology in the Young* 2017;**27**:677–85. doi:10.1017/S1047951116001025

For peer review only

BMJ Open

Ethics and legal requirements for data linkage in 14 European countries for children with congenital anomalies

Journal:	*BMJ Open*
Manuscript ID	bmjopen-2023-071687.R2
Article Type:	Communication
Date Submitted by the Author:	08-Mar-2023
Complete List of Authors:	Claridge, Hugh; University of Surrey, School of Health Sciences<br>Tan, Joachim; St George's University of London, Population Health Research Institute<br>Loane , Maria ; Ulster University Faculty of Life and Health Sciences<br>Garne, Ester; Lillebaelt Hospital - University Hospital of Southern Denmark, Department of Paediatrics and Adolescent Medicine<br>Barisic, Ingeborg; Children's Hospital Zagreb, Centre of Excellence for Reproductive and Regenerative Medicine, Medical School University of Zagreb<br>Cavero- Carbonell, Clara; Foundation for the Promotion of Health and Biomedical Research in the Valencian Region, Rare Diseases Research Unit<br>Dias, Carlos; National Institute of Health Doctor Ricardo Jorge, Epidemiology Department, National Registry of Congenital Anomalies<br>Gatt, Miriam; Directorate for Health Information and Research, Malta Congenital Anomalies Registry<br>Jordan, Susan; Swansea University, Faculty of Medicine, Health & Life Sciences<br>Khoshnood, Babak; University of Paris, Epidemiology and Statistics Research Center, Obstetrical, Perinatal and Pediatric Epidemiology Research Team, Institut National de la Santé et de la Recherche Médicale<br>Kiuru-Kuhlefelt, Sonja; Finnish Institute for Health and Welfare, Knowledge Brokers<br>Klungsoyr, Kari; University of Bergen, Department of Global Public Health and Primary Care; Norwegian Institute of Public Health, Divison of Mental and Physical Health<br>Mokoroa Carollo, Olatz; Biodonostia Health Research Institute, Public Health Division of Gipuzkoa<br>Nelen, Vera; Provincial Institute for Hygiene<br>Neville, Amanda; University of Ferrara, IMER Registry<br>Pierini, Anna; Institute of Clinical Physiology National Research Council<br>Randrianaivo, Hanitra; Centre Hospitalier Universitaire de la Reunion, Register of Congenital Malformations<br>Rissmann, Anke; Otto von Guericke University Medical Faculty, Malformation Monitoring Centre Saxony-Anhalt<br>Tucker, David; Public Health Wales NHS Trust<br>de Walle, Hermien; University of Groningen, University Medical Center Groningen, Department of Genetics<br>Wertelecki, Wladimir; OMNI-Net for Children International Charitable Fund<br>Morris, Joan; St George's University of London, Population Health

	Research Institute
**Primary Subject Heading**:	Ethics
Secondary Subject Heading:	Epidemiology, Public health
Keywords:	ETHICS (see Medical Ethics), EPIDEMIOLOGY, PUBLIC HEALTH, REGISTRIES

I, the Submitting Author has the right to grant and does grant on behalf of all authors of the Work (as defined in the below author licence), an exclusive licence and/or a non-exclusive licence for contributions from authors who are: i) UK Crown employees; ii) where BMJ has agreed a CC-BY licence shall apply, and/or iii) in accordance with the terms applicable for US Federal Government officers or employees acting as part of their official duties; on a worldwide, perpetual, irrevocable, royalty-free basis to BMJ Publishing Group Ltd ("BMJ") its licensees and where the relevant Journal is co-owned by BMJ to the co-owners of the Journal, to publish the Work in BMJ Open and any other BMJ products and to exploit all rights, as set out in our licence.

The Submitting Author accepts and understands that any supply made under these terms is made by BMJ to the Submitting Author unless you are acting as an employee on behalf of your employer or a postgraduate student of an affiliated institution which is paying any applicable article publishing charge ("APC") for Open Access articles. Where the Submitting Author wishes to make the Work available on an Open Access basis (and intends to pay the relevant APC), the terms of reuse of such Open Access shall be governed by a Creative Commons licence – details of these licences and which Creative Commons licence will apply to this Work are set out in our licence referred to above.

Other than as permitted in any relevant BMJ Author's Self Archiving Policies, I confirm this Work has not been accepted for publication elsewhere, is not being considered for publication elsewhere and does not duplicate material already published. I confirm all authors consent to publication of this Work and authorise the granting of this licence.

For peer review only

TITLE: Ethics and legal requirements for data linkage in 14 European countries for children with congenital anomalies

AUTHORS

Hugh Claridge, School of Health Sciences, University of Surrey, UK

Joachim Tan, Population Health Research Institute, St George's, University of London, UK

Maria Loane, Faculty of Life and Health Sciences, Ulster University, Belfast, UK

Ester Garne, Department of Paediatrics and Adolescent Medicine, Lillebaelt Hospital, University Hospital of Southern Denmark, Kolding, Denmark

Ingeborg Barisic, Children's Hospital Zagreb, Centre of Excellence for Reproductive and Regenerative Medicine, Medical School University of Zagreb, Croatia

Clara Cavero-Carbonell, Rare Diseases Research Unit, Foundation for the Promotion of Health and Biomedical Research in the Valencian Region (FISABIO), Valencia, Spain

Carlos Dias, Epidemiology Department, National Registry of Congenital Anomalies, National Institute of Health Doutor Ricardo Jorge (Instituto Nacional de Saúde Doutor Ricardo Jorge), Lisbon, Portugal

Miriam Gatt, Malta Congenital Anomalies Registry, Directorate for Health Information and Research, Pieta, Malta

Susan Jordan, Faculty of Medicine, Health & Life Sciences, Swansea University, Swansea, Wales

Babak Khoshnood, Université de Paris, CRESS-Epopé, INSERM, INRA, Paris, France

Sonja Kiuru-Kuhlefelt, Knowledge Brokers, Finnish Institute for Health and Welfare, Helsinki, Finland

Kari Klungsoyr, Department of Global Public Health and Primary Care, University of Bergen and Division of Mental and Physical Health, Norwegian Institute of Public Health, Bergen, Norway

Olatz Mokoroa Carollo, Public Health Division of Gipuzkoa, BioDonostia Research Institute, San Sebastian, Spain

Vera Nelen, Provincial Institute for Hygiene, Antwerp, Belgium

Amanda J Neville, Registro IMER, University of Ferrara, Ferrara, Emilia-Romagna, Italy

Anna Pierini, Institute of Clinical Physiology National Research Council, Pisa, Italy

Hanitra Randrianaivo, Register of Congenital Malformations, Centre Hospitalier Universitaire de La Réunion, Île de la Réunion, France

Anke Rissmann, Malformation Monitoring Centre Saxony-Anhalt, Medical Faculty Otto-von-Guericke University, Magdeburg, Germany

David Tucker, Public Health Wales National Health Service Trust, Wales, United Kingdom

Hermien de Walle, University of Groningen, University Medical Center Groningen, Department of Genetics, Groningen, the Netherlands

For peer review only - http://bmjopen.bmj.com/site/about/guidelines.xhtml

Wladimir Wertelecki, OMNI-Net for Children International Charitable Fund, Rivne, Ukraine

Corresponding author: Joan K. Morris, Population Health Research Institute, St George's, University of London, London, UK, SW17 0RE jmorris@sgul.ac.uk

WORD COUNT

1703 words

KEYWORDS

Ethics; Information Storage and Retrieval; Methods; Congenital Abnormalities; Rare Diseases; Europe

ABSTRACT

Introduction

Linking health care datasets can create valuable resources for research, particularly when investigating rare exposures or outcomes. However, across Europe the permissions processes required to access data can be complex. This paper documents the processes required by the EUROlinkCAT study investigators to research the health and survival of children with congenital anomalies in Europe.

Methods

Eighteen congenital anomaly registries in 14 countries provided information on all the permissions required to perform surveillance of congenital anomalies and to link their data on live births with available vital statistics and health care databases for research. Small number restrictions imposed by data providers were also documented.

Results

The permissions requirements varied substantially, with certain registries able to conduct congenital anomaly surveillance as part of national or regional health care provision, whilst others were required to obtain ethics approvals or informed consent. Data linkage and analysis for research purposes added additional layers of complexity for registries, with some required to obtain several permissions, including ethics approvals to link the data. Restrictions relating to small numbers often resulted in a registry's data on specific congenital anomalies being unusable.

Conclusion

The permissions required to obtain and link data on children with congenital anomalies varied greatly across Europe. The variation and complexity present a significant obstacle to the use of such data, especially in large data linkage projects. Furthermore, small number restrictions severely limited the research that could be performed for children with specific rare congenital anomalies.

For peer review only - http://bmjopen.bmj.com/site/about/guidelines.xhtml

INTRODUCTION

The EUROlinkCAT study aimed to support 22 congenital anomaly registries in 14 countries to link their data on live births with congenital anomalies to other regional or national electronic health care databases to investigate the survival and morbidity of these children up to ten years of age. This article briefly summarises the different legal, ethical, governance and other permissions that the registries had to obtain to perform this work.

METHODS

Each congenital anomaly registry was required to submit to the Coordinating Beneficiary of EUROlinkCAT (St George's, University of London), the permissions for the registry to fulfil its surveillance remit and to send data to EUROCAT (the European network of population-based congenital anomaly registries). An umbrella protocol was then supplied to each registry providing sufficient information for them to apply to the relevant authorities to enable them to link their data on liveborn children with congenital anomalies to national and regional electronic databases containing information on the survival and morbidity of these children. The registries were responsible for obtaining the necessary permissions, and once obtained these were submitted to the EUROlinkCAT Coordinator. This article summarises all the permissions obtained. As the permissions needed for surveillance differed from those needed for the subsequent research, details regarding the two sets of permissions are reported separately.

The English data on congenital anomalies included in the EUROlinkCAT project were provided by five regional congenital anomaly registries. However, in April 2015, the regional registries in England were all combined into the National Congenital Anomaly and Rare Disease Registration Service (NCARDRS). As the regional registries no longer exist, the permissions that would be required to obtain data from NCARDRS are reported here instead. This is why information is available for 18 registries in total, rather than the original 22.

Information from 18 registries in 14 countries are presented, relating to the timeframe of the EUROlinkCAT project from 2017-2022 – it is therefore possible that some permission requirements have since changed.

The answers from the registries were categorised in Table 1 under the following headings:

1. Permissions required for registry to perform surveillance of congenital anomalies
   - 1.1. Permission granted as part of regional/national health care provision
     - 1.1.1. Yes: National or regional legislation exists that enables the registry to collect data for surveillance purposes
     - 1.1.2. No: National or regional legislation does not exist to enable the registry to collect data for surveillance purposes
   - 1.2. Permissions from hosting institution

For peer review only - http://bmjopen.bmj.com/site/about/guidelines.xhtml

1.2.1. Yes: The registry needs to obtain permission to collect data for surveillance

1.2.2. No: No additional permission is needed as national/regional legislation is sufficient

1.3. Ethics permission

1.3.1. Yes: The registry needs to obtain ethics permission to collect data for surveillance

1.3.2. No: The registry does not need to obtain ethics permission to collect data for surveillance

1.4. Informed consent needed

1.4.1. Yes: The registry needs to obtain informed consent from the parents to register the congenital anomaly

1.4.2. No: The registry does not need to obtain informed consent from the parents to register the congenital anomaly. However, the parents can choose to have their details removed by contacting the relevant organisation.

2. Permissions required for registry to link data and analyse for research purposes

2.1. Ethics

2.1.1. Yes: Ethics permission is needed for research projects to link and analyse the linked data

2.1.2. No: Ethics permission is not needed for research projects using the linked data

2.2. Other permissions needed

2.2.1. Yes: Other permissions, not including ethics, such as approval by the data provider regarding the safety of the data storage, permissions to share the data with other named parties or permissions to release the analytic results for publication are required

2.2.2. No: No additional permissions are needed for research using the linked data

2.3. Small number restrictions

2.3.1. Yes-Pub: Small number restrictions apply to any publication of the data, but pre-publication results provided to named researchers may include small numbers

2.3.2. Yes-Int: Small number restrictions apply to all use of data including pre-publication results

2.3.3. No: No small number restrictions need to be applied.

For peer review only - http://bmjopen.bmj.com/site/about/guidelines.xhtml

RESULTS

Table 1: The permissions required for congenital anomaly registries to perform surveillance and research.

Full name of CA Registry (geographical region)	Permissions required for registry to perform surveillance of congenital anomalies				Permissions required for registry to link data and analyse for research purposes			Notes
	Permission granted as part of regional/national health care provision	Permission from hosting institution	Ethics permission	Informed consent	Ethics permission	Other Permissions	Small Number Restrictions	
**Antwerp Congenital Anomaly Registry** (Belgium: Antwerp)	Yes	No	No	No	Yes	Yes	Yes-Int	Considered linkage to mortality data only
**Zagreb Congenital Anomaly Registry** (Croatia: Zagreb)	No	Yes	No	No	Yes	No	No	
**Odense** (Denmark: Funen)	Yes	Yes	No	No	No	Yes	Yes-Pub	
**Finnish Register of Congenital Malformations** (Finland: all)	Yes	No	No	No	Yes	No	No	
**Registre des malformations congénitales de l'île de la Réunion** (France: Île de la Réunion)	Yes	No	Yes	No	Yes	Yes	No	
**Registre des malformations congénitales de Paris** (France: Paris)	Yes	No	Yes	No	Yes	Yes	No	Considered linkage to mortality data only
**Saxony-Anhalt Congenital Anomaly Registry** (Germany: Saxony-Anhalt)	No	No	Yes	Yes	Yes	No	No	Manual linkage only to mortality data due to Bundesstatistikgesetz $21 (Prohibition of re-identification)

For peer review only - http://bmjopen.bmj.com/site/about/guidelines.xhtml

**IMER Registry** (Italy: Emilia Romagna)	Yes	No	No	No	No	Yes	No	
**Tuscany Congenital Anomaly Registry** (Italy: Tuscany)	Yes	No	No	No	No	Yes	No	
**Malta Congenital Anomaly Registry** (Malta: all)	Yes	No	No	No	Yes	No	No	Considered linkage to mortality data only
**EUROCAT Registry - Northern Netherlands** (Netherlands: Northern)	Yes	No	No	Yes	Yes	Yes	Yes-Int	Other permissions: parents are given the option to opt out of EUROlinkCAT
**Medical Birth Registry of Norway** (Norway: All)	Yes	No	No	No	Yes	Yes	No	Considered linkage to mortality data only
**RENAC: Registo Nacional de Anomalias Congénitas** (Portugal: South)	Yes	Yes	No	No	Yes	Yes	No	
**Basque Congenital Anomaly Registry (Spain:** Basque Country)	Yes	No	No	No	Yes	No	No	
**Valencian Region Congenital Anomalies Registry** (Spain: Valencian Region)	Yes	No	No	No	Yes	No	No	
**National Congenital Anomaly and Rare Disease Registration Service (NCARDRS)** (UK: England)	Yes	No	No	No	Yes	Yes	Yes-Pub	NCARDRS became part of NHS Digital in October 2021 and access conditions are currently under review
**Congenital Anomaly Register and Information Service (CARIS) linked with SAIL databank** (UK: Wales)	Yes	No	Yes	No	No	Yes	Yes-Pub	

For peer review only - http://bmjopen.bmj.com/site/about/guidelines.xhtml

**OMNI-Net Ukraine Birth Defects Program** (Ukraine: West)	Yes	No	No	No	No	No	No	Considered linkage to mortality data only

For peer review only

Permissions required for surveillance of congenital anomalies

The majority (16/18) of registries have permission to perform surveillance of congenital anomalies as part of their national or regional health care provision. Of these 16, three are required to obtain ethics approval to perform this surveillance and one is required to obtain informed consent from the parents/guardians of the children. Of the two registries that did not have permission under their national or regional health care provision, one needed permission to collect the data from their host institution and one needed both ethics permission and informed consent.

Permissions required for data linkage and analysis for research purposes

Of the six registries who only linked their data to mortality data, five required specific ethics permission to do so, and three of these also required other additional permissions. Of the 12 registries who linked their data to electronic health care databases, eight required specific ethics permission to do so and four of these required other additional permissions. The four registries who did not require specific ethics permission to link their data to electronic health care databases did however require other additional permissions, such as demonstrating to the data providers that the data would be stored safely, that the project was of sufficient scientific merit, and other assurances arising from the European Union's General Data Protection Regulation (GDPR) 2016/679 requiring that the data be used in a transparent manner.

Once the data had been linked to other databases, the authorities providing these data often imposed strict restrictions on the publication of results when small numbers of children were involved. This was to ensure that children with rare congenital anomalies and their families could not be identified. Some authorities did allow aggregate results from small numbers of individuals to be distributed amongst named researchers with the requirement that these results could only be published when combined with data from other registries. Permitting the internal distribution amongst specified researchers was extremely helpful as it enabled meta-analyses over several European countries to be performed for groups of rare congenital anomalies and combined results to be published. Authorities suppressing the provision of small numbers of cases for analysis (e.g. through rounding counts to the nearest 5) often resulted in a registry's data being excluded from the meta-analyses due to the rounding having a significant effect on the overall estimates.

The length of time required to obtain the necessary permissions varied considerably by registry, with several registries taking over two years to obtain the permissions and thus over two years to obtain the final linked data. In contrast, some institutions granted approvals within three months.

DISCUSSION

To the authors' knowledge, this is the first example in the literature of a multi-centre, multi-national health data linkage project documenting the broad range of permissions required across Europe to access and link data on children with congenital anomalies. The increased awareness that this article provides of the high variability in data providers' requirements and the timescales that the

For peer review only - http://bmjopen.bmj.com/site/about/guidelines.xhtml

permissions-related processes operate within, will be of great assistance to funding bodies and both current and future studies in this and related fields. For multi-centre studies, the widely variable waiting times make it very challenging to determine a common timeframe for the overall study to work to. Indeed, the challenges faced by EUROlinkCAT researchers are not unique, with researchers across the world facing similar issues (UK:[1]; Australia:[2]; USA:[3]). Advanced knowledge of the challenges means staff time and thus personnel budgets can be more accurately costed over a project's timeframe; awareness of the possibility of permissions inter-dependencies can permit improved planning and more efficient task prioritisation; data access expiration dates can be more appropriately chosen to limit the need for amendments and re-applications; and staff can be better prepared for the sometimes demoralising wait for permissions to be granted.

The suppression of small numbers, such as preventing the release of numbers smaller than 10 even for internal analysis purposes, has a seriously detrimental impact on rare disease research. To illustrate this problem, consider Ebstein's Anomaly, which is a severe rare congenital heart defect with a mortality of around 50% in the first month of life,[4] and continued high mortality in the first five years of life.[5] The prevalence of Ebstein's Anomaly is about 4 per 100,000 births.[6] EUROlinkCAT registries have survival information on about 400 live births with Ebstein's Anomaly. If the numbers of children dying during the first month of life, the numbers dying after the first month of life and before their first birthday, and the numbers surviving past the age of one are required, all 22 EUROlinkCAT registries would be likely to have at least one out of the three cells below 10 and therefore none of the registries would be able to provide any aggregated data. Hence, no information about mortality during the first year of life could be obtained, despite a sample size of 400 children from a study period of 20 years and a population of 10 million births.

The rationale for small number restrictions is to prevent individuals or their families being identified. A distinction needs to be made between data released for publication and data released to named researchers for analysis. The implementation of GDPR across Europe in 2018 has ensured that all European countries have structures in place to ensure the safe use of an individual's data. These regulations should be applied in such a way as to allow all European countries to provide aggregate data to be used for pan-European analyses across countries to trusted, named researchers, even if certain categories of such aggregate data comprise fewer than 10 cases.

CONCLUSIONS

There is a lack of consistency across Europe in the permissions required to access and link data on children with congenital anomalies. The complexity and time taken to obtain such permissions present a significant obstacle to the use of such data. In addition, the imposition of small number restrictions has a detrimental effect on the analyses that can be performed for children with rare anomalies.

For peer review only - http://bmjopen.bmj.com/site/about/guidelines.xhtml

AUTHORS' CONTRIBUTIONS

JKM is the principal author and the project's scientific coordinator. HC is the senior project manager. JT is the senior statistician. ML is the data coordinator. EG is the clinical coordinator and EUROCAT registry leader. IB, CC-C, CD, MG, SJ, BK, SK-K, KK, OMC, VN, AN, AP, HR, AR, DT, HdW and WW are EUROCAT registry leaders. All authors contributed to, read and approved the final manuscript.

FUNDING STATEMENT

This project has received funding from the European Union's Horizon 2020 Research and Innovation programme under grant agreement No 733001 for 5 years: 1 January 2017 to 31 May 2022. The views presented here are those of the authors only, and the European Commission is not responsible for any use that may be made of the information presented here.

COMPETING INTERESTS STATEMENT

The authors have no competing interests to declare.

For peer review only

For peer review only - http://bmjopen.bmj.com/site/about/guidelines.xhtml

REFERENCES

1 Taylor JA, Crowe S, Pujol FE, *et al.* The road to hell is paved with good intentions: the experience of applying for national data for linkage and suggestions for improvement. *BMJ Open* 2021;**11**:e047575. doi:10.1136/bmjopen-2020-047575

2 Palamuthusingam D, Johnson DW, Hawley C, *et al.* Health data linkage research in Australia remains challenging. *Internal Medicine Journal* 2019;**49**:539–44. doi:10.1111/imj.14244

3 Bradley CJ, Penberthy L, Devers KJ, *et al.* Health Services Research and Data Linkages: Issues, Methods, and Directions for the Future. *Health Services Research* 2010;**45**:1468–88. doi:10.1111/j.1475-6773.2010.01142.x

4 McElhinney DB, Salvin JW, Colan SD, *et al.* Improving Outcomes in Fetuses and Neonates With Congenital Displacement (Ebstein's Malformation) or Dysplasia of the Tricuspid Valve. *The American Journal of Cardiology* 2005;**96**:582–6. doi:10.1016/j.amjcard.2005.04.024

5 Holst KA, Dearani JA, Said SM, *et al.* Surgical Management and Outcomes of Ebstein Anomaly in Neonates and Infants: A Society of Thoracic Surgeons Congenital Heart Surgery Database Analysis. *The Annals of Thoracic Surgery* 2018;**106**:785–91. doi:10.1016/j.athoracsur.2018.04.049

6 Boyle B, Garne E, Loane M, *et al.* The changing epidemiology of Ebstein's anomaly and its relationship with maternal mental health conditions: a European registry-based study. *Cardiology in the Young* 2017;**27**:677–85. doi:10.1017/S1047951116001025

For peer review only

For peer review only - http://bmjopen.bmj.com/site/about/guidelines.xhtml
